# Comparing outcomes for retrograde intramedullary nailing vs. antegrade intramedullary nailing for Femoral fractures – a systematic review and meta-analysis

**DOI:** 10.1051/sicotj/2026030

**Published:** 2026-05-26

**Authors:** Kapilraj Ravendran, Mohammed Ibrahim Khalil, Thiviya Manoharan, Achsah Ann Alexander, Nuraan Shahid, Karim Abdelghafour, Pranav Mishra

**Affiliations:** 1 East and North Hertfordshire NHS Trust Stevenage UK; 2 Gradscape London UK; 3 Medical University Plovdiv Bulgaria; 4 Northern Care Alliance NHS Foundation Trust UK; 5 Tamesdie and Glossop Integrated Care NHS Foundation Trust UK

**Keywords:** Antegrade approach, Retrograde approach, Femoral fracture

## Abstract

*Background*: Distal femur fractures represent a small but clinically significant proportion of femoral injuries and are associated with high rates of complications, including non-union and revision surgery. Intramedullary nailing is a commonly employed fixation strategy, with both antegrade and retrograde approaches widely used in current practice. However, controversy persists regarding the optimal nail entry technique, particularly with respect to fracture healing, revision rates, and complications. This systematic review and meta-analysis aimed to compare clinical and radiological outcomes of antegrade versus retrograde intramedullary nailing for femoral shaft and distal femur fractures. *Methods*: This review was conducted in accordance with PRISMA guidelines and registered with PROSPERO (CRD420251274011). MEDLINE, EMBASE, and Cochrane databases were systematically searched for comparative studies evaluating antegrade and retrograde intramedullary nailing. Retrospective and prospective human studies reporting outcomes of union, revision, refracture, malunion, delayed union, operative time, and complications were included. Data extraction and quality assessment were independently performed, with risk of bias evaluated using the ROBINS-I tool. Meta-analysis was conducted using odds ratios (ORs) and weighted mean differences with 95% confidence intervals. *Results*: Five studies encompassing 1,479 patients were included, of whom 894 underwent antegrade nailing, and 585 underwent retrograde nailing. Antegrade nailing demonstrated a significantly lower rate of refracture following primary fixation compared with retrograde nailing (OR 31.41; 95% CI 4.45–221.80; *p* < 0.001). Revision rates were also significantly lower in the antegrade group (OR 1.76; 95% CI 1.21–2.58; *p* = 0.003). Retrograde nailing showed a higher overall union rate, although this did not reach statistical significance (*p* = 0.10). Rates of malunion, delayed union, non-union, operative time, and overall complications were comparable between groups, with moderate to substantial heterogeneity observed for several outcomes. *Conclusion*: Both antegrade and retrograde intramedullary nailing provide effective fixation for femoral shaft and distal femur fractures. Antegrade nailing is associated with significantly lower rates of refracture and revision, while retrograde nailing demonstrates comparable union outcomes. These findings suggest that antegrade nailing may offer advantages in selected patient populations, particularly elderly individuals, although the surgical approach should ultimately be guided by fracture pattern, patient factors, and surgeon expertise.

## Introduction

Distal femur fractures account for 0.4% of all fractures and 3–6% of femur fractures [1]. Its age distribution is bimodal, with young people (20–30 years old with high energy injuries such as road traffic accidents) and elderly women (approximately 70 years old, with low energy injuries such as falls from standing height) being the most common [2, 3]. The goals of surgical fixation for these fractures are relative stability for diaphyseal fractures to initiate early mobilization, anatomical articular reduction, and blood supply preservation [4]. The non-union rate is quoted as high as 18–20% [5].

Compared to an eccentrically positioned plate and screws construct, intramedullary nails are load-sharing devices that stay close to the femoral axis and have better stress distribution. Nailing also requires a shorter operating time, less perioperative blood loss, and early patient mobilization [6].

One proven treatment for these fractures is retrograde nailing [7]. Prior research on the results of patients treated with retrograde nailing frequently included both young individuals with high-energy fractures and older patients with osteoporotic fractures [8, 9]. Retrograde intramedullary nailing was first used by Green in 1988 to treat distal femur fractures [10]. Its benefits include indirect reduction and internal fixation, which prevent excessive soft tissue dissection, periosteal blood supply interruption, and provide high union rates [11, 12].

Antegrade intramedullary nailing of the femur is an effective treatment for diaphyseal fractures [13]. The antegrade approach to femoral nailing is the favored way of fixation for the majority of surgeons due to its relative simplicity of patient posture (supine position with or without ipsilateral traction) and obvious surgical starting locations (piriformis fossa or greater trochanter, depending on nail design) [14].

A key consideration in antegrade intramedullary nailing is protection of the femoral neck, particularly in older osteoporotic patients, in whom reduced bone mineral density increases the risk of iatrogenic femoral neck fracture during entry point creation, reaming, or nail insertion. Malposition of the entry point, excessive insertion force, and implant–canal mismatch may increase stress within the proximal femur, predisposing to fracture. Given the substantial biomechanical forces transmitted through the femoral neck, meticulous surgical technique, appropriate implant selection, and careful intraoperative imaging are essential to minimize this risk [5].

The two methods for treating distal femur fractures – antegrade and retrograde intramedullary nailing – are compared and contrasted in this systematic study.

### Search strategy and study design

A study protocol was developed and agreed upon by all authors prior to commencement. The review was conducted in accordance with the Preferred Reporting Items for Systematic Reviews and Meta-Analyses (PRISMA) 2020 guidelines [15]. A comprehensive electronic search was performed across MEDLINE (via PubMed), EMBASE, and the Cochrane Library, including the Cochrane Central Register of Controlled Trials.

Search terms were constructed using combinations of keywords and Medical Subject Headings (MeSH), including: (“femoral shaft fracture” OR “distal femur fracture”) AND (“antegrade intramedullary nailing” OR “retrograde intramedullary nailing”) AND (“union” OR “non-union” OR “revision” OR “malunion” OR “post-operative fracture”). Reference lists of included studies were manually screened to identify additional relevant articles.

### Eligibility criteria

Studies were eligible for inclusion if they directly compared antegrade and retrograde intramedullary nailing for femoral shaft or distal femur fractures and reported at least one predefined clinical or radiological outcome. Both retrospective and prospective studies involving human participants were included. Exclusion criteria comprised biomechanical or cadaveric studies, case reports, narrative reviews, editorials, non-English publications, and studies lacking full-text availability.

Primary outcomes were fracture union, revision surgery, and refracture following index fixation. Secondary outcomes included operative time, mobility-related outcomes, and overall complication rates. The review was prospectively registered with PROSPERO (CRD420251274011).

### Data extraction and quality assessment

Data extraction was independently undertaken by four reviewers (M.I.K., A.S., T.M., and N.S.) using a standardized extraction form. Extracted data were cross-checked by senior authors (K.R., K.A., and P.M.) to ensure accuracy. Information collected included study characteristics, patient demographics, fracture type, surgical technique, follow-up duration, and reported outcomes.

Risk of bias was assessed independently by two reviewers (K.R. and P.M.) using the ROBINS-I tool for non-randomized studies [16]. Discrepancies were resolved by consensus. Risk-of-bias visualization was generated using the ROBVIS tool [17].

### Statistical analysis

Meta-analyses were conducted using Review Manager (RevMan version 5.4). Dichotomous variables were pooled using odds ratios (ORs), while continuous outcomes were analyzed using weighted mean differences, both reported with 95% confidence intervals. Statistical heterogeneity was evaluated using the Chi-square test and quantified with the I^2^ statistic, with values exceeding 50% indicating substantial heterogeneity. Publication bias was assessed through funnel plot analysis and Egger’s regression test.

## Results

**Figure 1:**
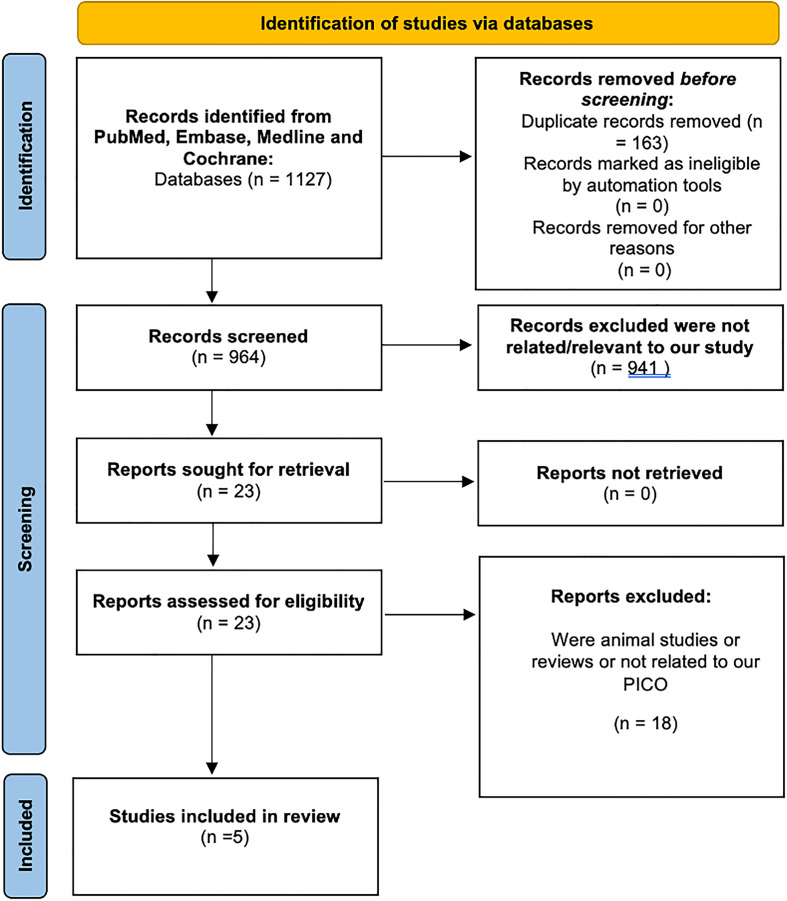
PRISMA: Preferred reporting items for systematic reviews and meta-analyses.

In the initial database search, 1127 items were found; 163 of them were removed due to duplication. Following abstract screening and the application of the exclusion criteria, 941 of the remaining ones were eliminated. Eighteen were deleted after the entire texts were evaluated to determine their relevance to the review subject. The final evaluation had five papers in total. A PRISMA-style graphic depicting the book selection procedure is shown in Figure 1 [15].

Five hundred and eighty-five of the five studies used retrograde intramedullary nailing, whereas 894 used antegrade intramedullary nailing [18–22]. The research comprised populations from Sweden, Turkey, Malaysia, Nigeria, and the United States [18–22].

Study characteristics are demonstrated in Table 1 [18–22].

**Table 1:** Study characteristics of included studies.

Author (Year)	Study period	Country	Age – Retrograde	Age – Antegrade	Sex – Retrograde (F/M)	Sex – Antegrade (F/M)
Adesina et al. (2023) [18]	2014–2022	Nigeria	39.58 (NR)	35.44 (NR)	54 / 100	25 / 59
Gönder et al. (2023) [19]	2016–2021	Turkey	49.93 ± 18.31	46.22 ± 18.60	21 / 19	19 / 15
Ricci et al. (2001) [20]	1993–1997	USA	34 (NR)	32 (NR)	27 / 72	29 / 65
Chan et al. (2007) [21]	2004–2005	Malaysia	37.4 (NR)	42 (NR)	7 / 28	6 / 36
Bogl et al. (2020) [22]	2008–2014	Sweden	Median 80.4 (70.8–87.4)	Median 84.3 (77.5–88.8)	226 / 31	504 / 136

Clinical and radiological findings are summarized in Table 2 [18–22].

**Table 2:** Clinical and radiological outcomes (Retrograde vs Antegrade).

Author	Refracture R	Refracture A	Reoperation R	Reoperation A	Union R	Union A	Malunion R	Malunion A	Delayed union R	Delayed union A	Non-union R	Non-union A	Union after revision R	Union after revision A	Mobility / function R	Mobility / function A	Op time R (min)	Op time A (min)	Complications R	Complications A
Adesina et al. [18]	*	*	*	*	*	*	*	*	*	*	*	*	*	*	12-wk recovery 132; KF>90° 141; WB 141	12-wk recovery 77; KF>90° 80; WB 81	63.93 ± 39.60	84.04 ± 34.30	2 infections	1 infection
Gönder et al. [19]	*	*	*	*	*	*	*	*	2	1	*	*	*	*	Sanders 29.7 ± 5.85; Lysholm 70.6 ± 10.6	Sanders 30.1 ± 5.63; Lysholm 81.4 ± 7.38	88.75 ± 14.49	77.92 ± 13.33	7 (IF 3, INF 4)	4 (INF)
Ricci et al. [20]	3	0	26	22	91 (88%)	84 (89%)	11 (11%)	12 (13%)	7 (7%)	4 (4%)	6 (6%)	6 (6%)	101 (97%)	93 (99%)	NR	NR	86–118	94–116	51 (knee pain)	55 (HO 26%)
Chan et al. [21]	0	0	0	0	35 (100%)	36 (85.7%)	0	0	0	4 (9.5%)	0	2 (4.8%)	42 (100%)	42 (100%)	Good–excellent flexion 91.4%	Good–excellent flexion 88.1%	NR	NR	1 knee pain	9 total
Bogl et al. [22]	14 proximal	0	36 total	46 total	*	*	*	*	*	*	*	*	*	*	NR	NR	NR	NR	14 proximal peri-implant	7 distal peri-implant

R = Retrograde; A = Antegrade; NR = Not reported; * = Not stratified by approach; KF = knee flexion; WB = weight bearing; IF = implant failure; INF = infection; HO = heterotopic ossification.

Qualitative findings are summarized in Table 3 [18–22].

**Table 3:** Qualitative data (Retrograde vs Antegrade).

Author	Fractture type	Number of each fracture type	Cause of refracture	Reason for reoperation	Reported complications
Adesina et al. [18]	Diaphyseal femur fractures (AO/OTA type 32A, 32B, 32C)	32-A: Retrograde 63; Antegrade 43; Total 106 32-B: Retrograde 69; Antegrade 33; Total 102 32-C: Retrograde 22; Antegrade 8; Total 30	NR	NR	Antegrade – Infection – 1 Retrogarde Infection – 2
Gönder et al. [19]	Extra-articular distal femur fractures (AO/OTA 33A2–33A3) and distal femoral shaft fractures (AO/OTA 32A1c, 32A2c, 32A3c, 32B2c, 32B3c) including distal diaphyseal–metaphyseal fractures (32C3k).	33A2: Retrograde 19; Antegrade 11 33A3: Retrograde 13; Antegrade 4 32A1c: Retrograde 2; Antegrade 5 32A2c: Retrograde 3; Antegrade 1 32A3c: Retrograde 1; Antegrade 3 32B2c: Retrograde 3; Antegrade 7 32B3c: Retrograde 5; Antegrade 4 32C3k: Retrograde 2; Antegrade 1	NR	NR	Antegrade – Implant failure = 0 Non-union = 1 Infection = 4 Retrograde Implant failure = 3 Non-union = 2 Infection = 4
Ricci et al. [20]	Femoral diaphyseal shaft fractures. Categorized by location (proximal/middle/distal), open vs closed injury, degree of comminution (Winquist I–IV), and OTA types (32A–C).	Proximal one third: 31 total (10 in Group R; 21 in Group A).Middle one third: 111 total (52 in Group R; 59 in Group A).Distal one third: 56 total (42 in Group R; 14 in Group A). Closed: 154 total (80 in Group R; 74 in Group A). Open: 44 total (24 in Group R; 20 in Group A). Winquist Type I: 38 (Group R), 41 (Group A. Winquist Type II: 23 (Group R), 15 (Group A). Winquist Type III: 26 (Group R), 20 (Group A). Winquist Type IV: 17 (Group R), 18 (Group A). OTA Type 32A: 38 (Group R), 42 (Group A). OTA Type 32B: 51 (Group R), 34 (Group A). OTA Type 32C: 15 (Group R), 18 (Group A).	Antegrade – 0 Retrograde nailing – 3 Refractures. (1 – nail fracture, 1 – ipsilateral coronal fracture of medial femoral condyle after knee arthroscopy treated with ORIF, 1 – ipsilateral patella fracture).	Antegrade nailing – 22 – in total. 16 for mechanical / hardware issues. 6 additional revision procedures for delayed union/nonunion. (5 – Removal of painful interlocking screws, 4 – nail revision for shortening, 4 – nail removals, 1 – exchange of loose interlocking screw, 1 – revision of bent nail, 1 – removal of exostosis, 1 – dynamization for delayed union, 5 – exchange nail reoperations for nonunion). Retrograde nailing – 26 – in total 17 for mechanical or knee-related issues. 9 additional revision procedures for delayed/nonunion. (6 – Removal of painful interlocking screws, 4 – knee arthroscopy, 2 – knee manipulations, 1 – rotational malalignment, 1 – nail fracture, 1 – nail removal after migration to knee, 1 – removal of heterotopic bone from post-patellar region, 1 – aborted retrograde nailing and revised with antegrade nailing, 5 – exchange nail reoperations for nonunion, 3 – dynamizations for delayed union, 1 – dynamization for nonunion).	Antegrade Knee pain – 11 (12%) Hip pain – 10 (10%) Fractured interlocking screws – 4 (4%) Bent nail – 1 Heterotopic ossification – 24 (26%) Pulmonary embolism – 2 (2%) Fat embolus syndrome – 1 (1%) Deep venous thrombosis – 1 (1%) Transient pudendal nerve palsy – 1 (1%) 84 Unions – 89% 4 Delayed unions – 4% 12 Malunions – 13% 6 Nonunions – 6% 93 Unions after revisions – 99% Retrograde: - Knee pain – 34 (33%) Hip pain – 3 (4%) Fractured interlocking screws – 9 (9%) Nail Migration into knee joint – 2 Nail Fracture – 1 Heterotopic ossification – 1 Rotational Malalignment – 1 91 Unions – 88% 7 Delayed unions – 7% 11 Malunions – 11% 6 Nonunions – 6% 101 Unions after revisions – 97%
Chan et al. [21]	Femoral diaphyseal shaft fractures. Categorized by location (proximal/middle/distal), open vs closed injury, and degree of comminution (Winquist I–IV).	Proximal: 13 (Antegrade), 0 (Retrograde). Middle: 25 (Antegrade), 10 (Retrograde). Distal: 4 (Antegrade), 24 (Retrograde). Closed: 36 (Antegrade), 30 (Retrograde). Open (Grade 1 & 2): 5 (Antegrade), 4 (Retrograde). Open (Grade 3): 1 (Antegrade), 0 (Retrograde). Winquist Type 1: 16 (Antegrade), 21 (Retrograde). Winquist Type 2: 6 (Antegrade), 7 (Retrograde). Winquist Type 3: 7 (Antegrade), 5 (Retrograde). Winquist Type 4: 13 (Antegrade), 1 (Retrograde).	Antegrade – 0 Retrograde – 0	Antegrade – 0 Retrograde – 0	Antegrade Knee pain – 9.5% of patients. Implant Infection – 1 case. Implant failure – 3 cases. Malalignment – 2 – 4 cases. 36 Unions – 85.7% 4 Delayed Unions – 9.5% 2 Nonunions - 4.8% Malunion – not significant 42 Unions after revisions – 100% Retrograde Knee pain – 2.9% of patients. Union – 100% – in all 35 cases
Bogl et al. [22]	low-energy femoral shaft (diaphyseal) fractures and subtrochanteric fractures (proximal metaphyseal/transition zone fractures).	Diaphyseal (Shaft): 134 (Femoral Neck Protection), 248 (No Protection). Subtrochanteric: 506 (Femoral Neck Protection), 9 (No Protection).	Antegrade – 0 Retrograde -: 14 proximal peri-implant hip fractures	Antegrade – 27 major (implant revision/removal/arthroplasty) + 19 minor (hardware/wound revision) Retrograde – 24 major (implant revision/removal/arthroplasty) + 12 minor (hardware/wound revision)	Antegrade 7 distal peri implant fractures, no proximal fractures or avascular necrosis reported Retrograde 14 proximal peri implant hip fractures and no avascular necrosis reported

### Refracture after primary operation

**Figure 2:**
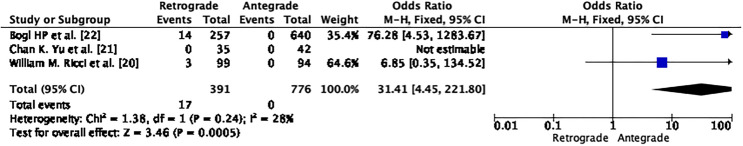
Demonstrates forrest graph for refracture after primary operation.

According to our research, the antegrade approach had a significantly lower rate of fracture after primary fixation compared to the retrograde approach, with a moderate heterogeneity (OR 31.41; CI – 4.45 – 221.80; *p* = 0.0.0005; I2 – 28%) as seen in Figure 2.

**Figure 3:**
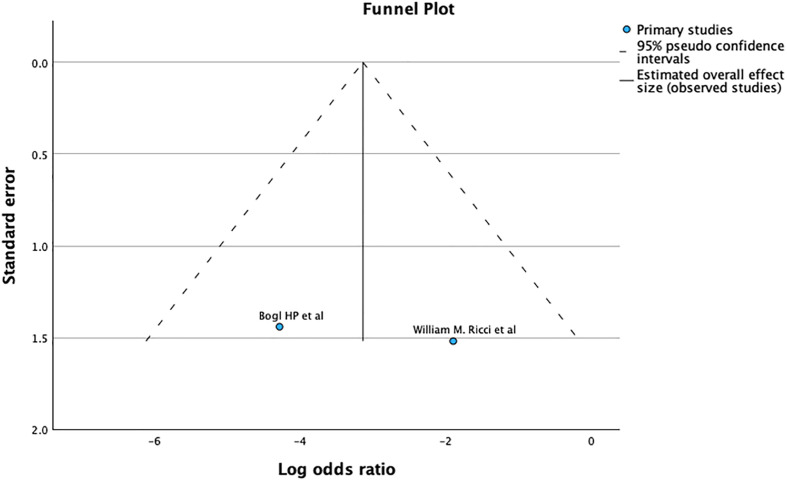
Demonstrates Funnel plot and Egger’s test for refracture after primary fixation. Predictor – Standard error *Z* = −2.632, *p* = 0.008 CI: −5.470 to −0.801.

The funnel plot and Egger’s test (*p* = 0.005), as shown in Figure 3, suggest significant publication bias. A meta-analysis of I2 – 28% shows Moderate heterogeneity.

### Revision

**Figure 4:**
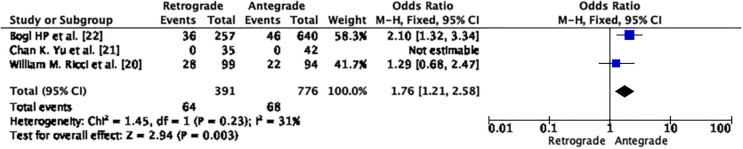
Demonstrates forrest graph for revision after primary fixation.

According to our research, the antegrade approach had a significantly lower rate of revision after primary fixation compared to the retrograde approach, with a moderate heterogeneity (OR 1.76; CI – 1.21 – 2.58; *p* = 0.0.003; I2 – 31%) as seen in Figure 4.

**Figure 5:**
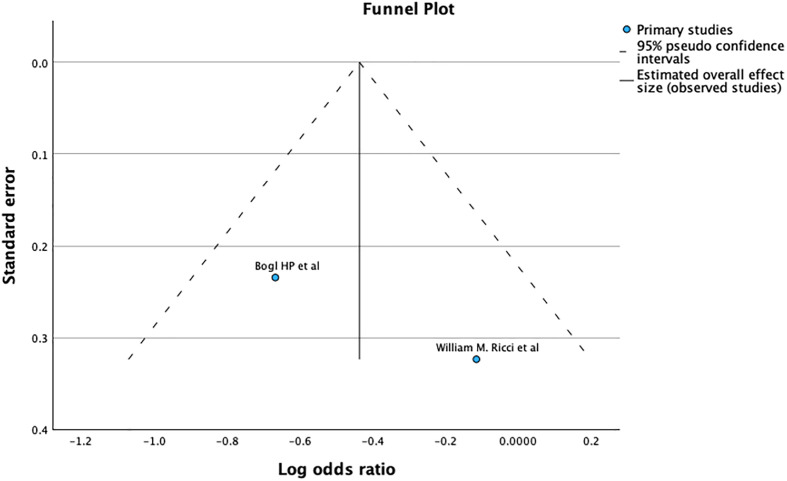
Demonstrates Funnel plot and Egger’s test for revision after primary fixation. Predictor – Standard error *Z* = −1.602, *p* = 0.109 CI: −0.970 – 0.098.

The funnel plot and Egger’s test (*p* = 0.109), as shown in Figure 5, suggest no significant publication bias. A meta-analysis of I2 31% shows Moderate heterogeneity.

### Union

**Figure 6:**
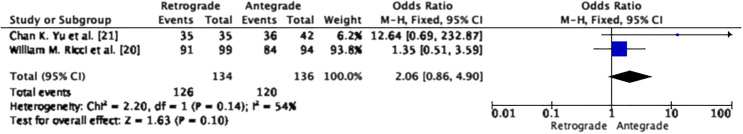
Demonstrates forrest graph for union after primary fixation.

According to our research, the antegrade approach had a lower rate of union after primary fixation compared to the retrograde approach (OR 2.06; CI – 0.86 – 4.90; *p* = 0.10; I2 – 54%) as seen in Figure 6.

**Figure 7:**
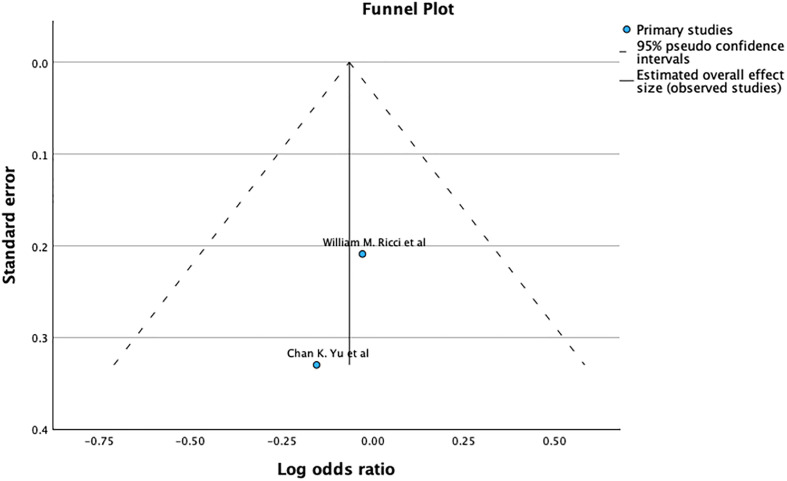
Demonstrates Funnel plot and Egger’s test for union after primary fixation. Predictor – Standard error *Z* = −0.364, *p* = 0.716 CI: −0.410 – 0.282.

The funnel plot and Egger’s test (*p* = 0.716), as shown in Figure 7, suggest no significant publication bias. A meta-analysis of I2 – 54% shows Substantial heterogeneity.

### Malunion

**Figure 8:**
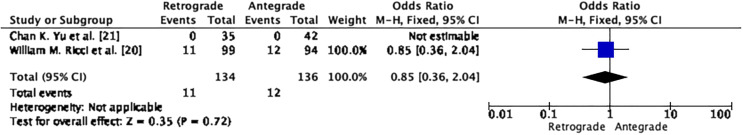
Demonstrates forrest graph for malunion after primary fixation.

According to our research, the antegrade approach had a higher rate of malunion after primary fixation compared to the retrograde approach (OR 0.85; CI – 0.36 – 2.04; *p* = 0.72; I2 – N/A) as seen in Figure 8.

**Figure 9:**
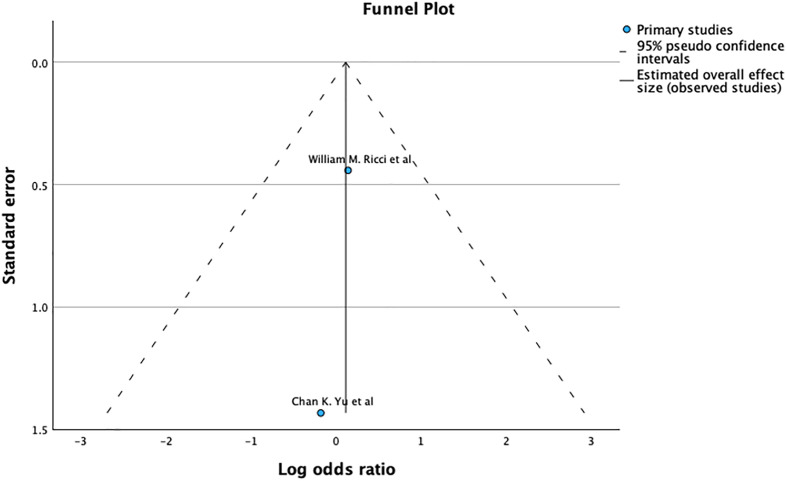
Demonstrates Funnel plot and Egger’s test for malunion after primary fixation. Predictor – Standard error *Z* = −2.63, *p* = 0.793 CI: −0.716 – 0.938.

The funnel plot and Egger’s test (*p* = 0.793), as shown in Figure 9, suggest no significant publication bias.

### Delayed union

**Figure 10:**
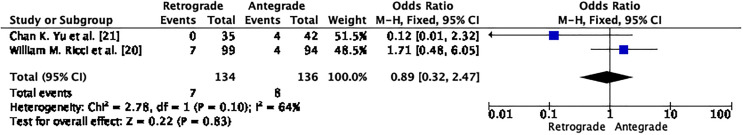
Demonstrates forrest graph for delayed union after primary fixation.

According to our research, the antegrade approach had a higher rate of delayed union after primary fixation compared to the retrograde approach (OR 0.89; CI – 0.32 – 2.47; *p* = 0.83; I2 – 64%) as seen in Figure 10.

**Figure 11:**
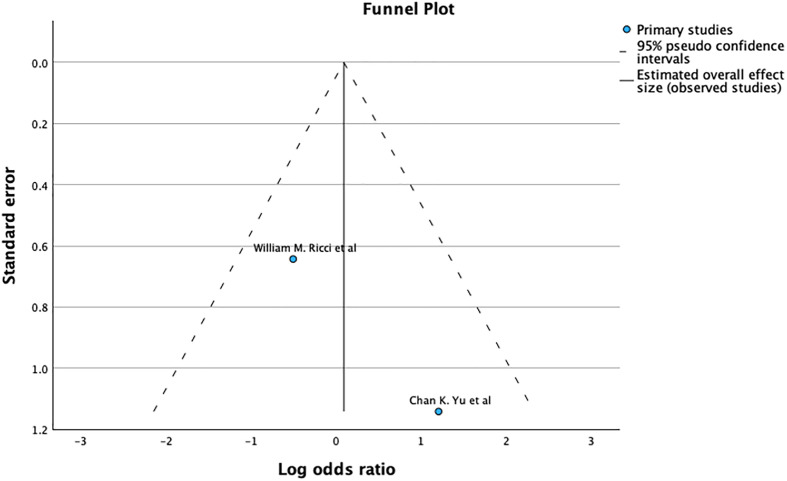
Demonstrates Funnel plot and Egger’s test for delayed union after primary fixation. Predictor – Standard error *Z* = 0.108, *p* = 0.914 CI: −1.510 – 1.687.

The funnel plot and Egger’s test (*p* = 0.914), as shown in Figure 11, suggest no significant publication bias. A meta-analysis, *I*^2^ = 54% shows Substantial heterogeneity.

### Non-union

**Figure 12:**
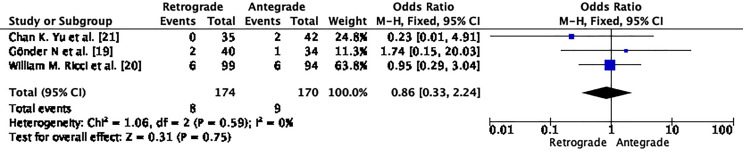
Demonstrates forrest graph for non-union after primary fixation.

According to our research, the antegrade approach had a higher rate of non-union after primary fixation compared to the retrograde approach (OR 0.86; CI – 0.33 – 2.24; p=0.75; I2 – 0%) as seen in Figure 12.

**Figure 13:**
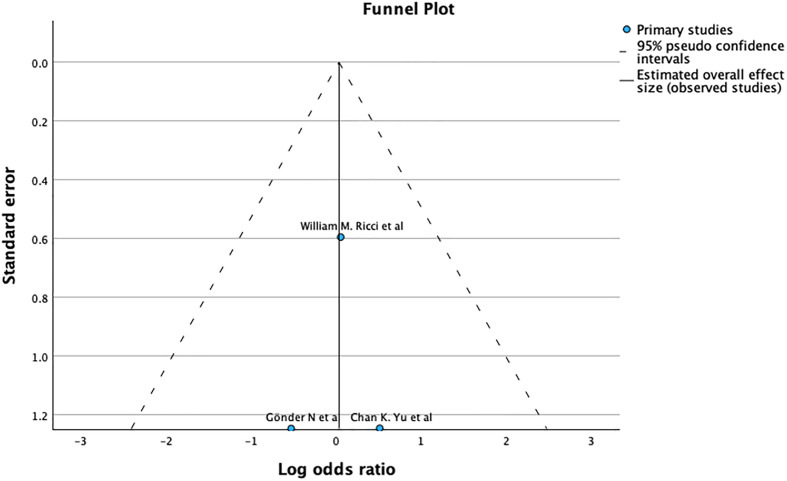
Demonstrates Funnel plot and Egger’s test for non-union after primary fixation. Predictor – Standard error *Z* = 0.066, *p* = 0.947 CI: −0.934 – 0.999.

The funnel plot and Egger’s test (*p* = 0.947), as shown in Figure 13, suggest no significant publication bias. A meta-analysis of I2 – 0%.

### Operation time

**Figure 14:**
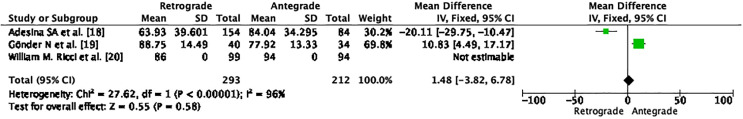
Demonstrates forrest graph for mean operative time.

According to our research, the antegrade approach had similar operative time compared to the retrograde approach (OR 1.48; CI: −3.82 to 6.78; *p* = 0.58; I2 – 96%) as seen in Figure 14.

**Figure 15:**
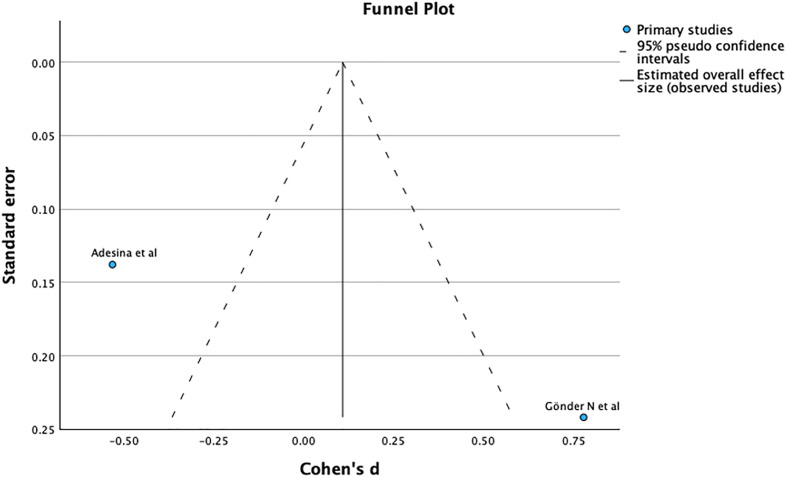
Demonstrates Funnel plot and Egger's test for mean operative time. Predictor. Standard error *Z* = 0.165, *p* = 0.869 CI: −1.175 – 1.391.

The funnel plot and Egger’s test (*p* = 0.869), as shown in Figure 15, suggest no significant publication bias. A meta-analysis of I2 – 96%.

### Complications

**Figure 16:**
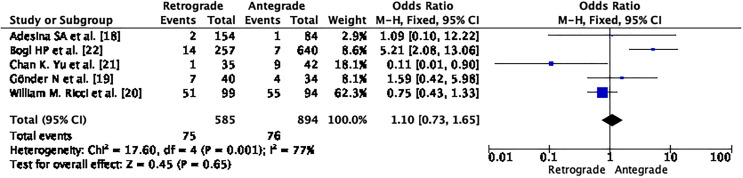
Demonstrates forrest graph for complications after primary fixation.

According to our research, the antegrade approach had a lower incidence of complications after primary fixation compared to the retrograde approach (OR 1.10; CI – 0.73 – 1.65; *p* = 0.65; I2 – 77%) as seen in Figure 16.

**Figure 17:**
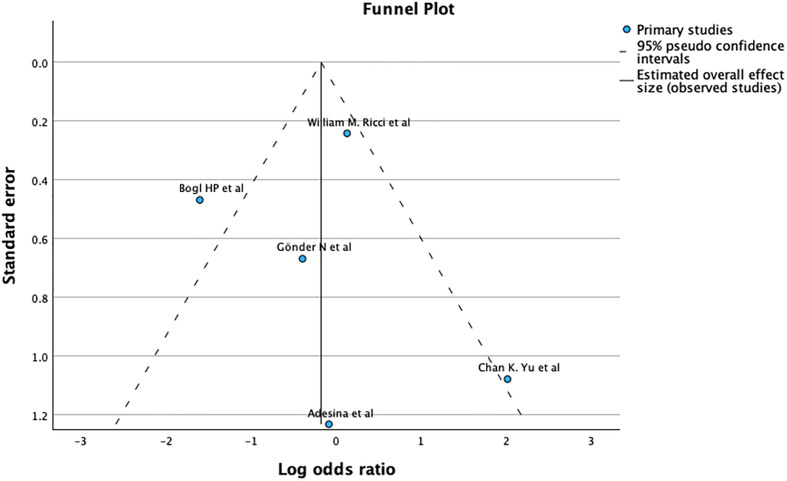
Demonstrates Funnel plot and Egger’s test for complications after primary fixation. Predictor – Standard error. *Z* = −0.330, *p* = 0.742 CI: −1.235 – 0.880.

The funnel plot and Egger’s test (*p* = 0.742), as shown in Figure 17, suggest no significant publication bias. A meta-analysis of I2 – 77% – shows Considerable heterogeneity.

## Data quality

**Figure 18:**
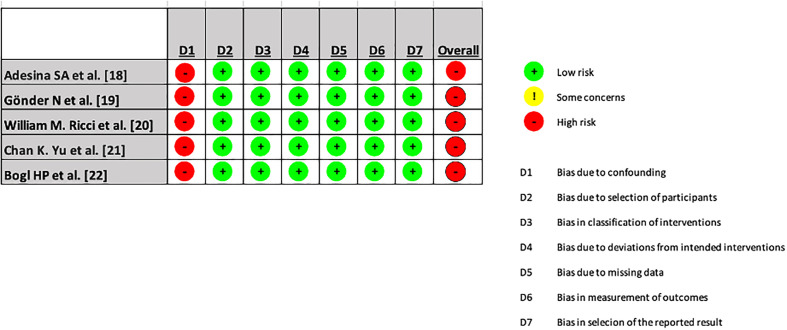
ROBINS I ROBVIS tool – Each study included in the meta-analysis is evaluated separately in Figure 18.

All of the included studies were either retrospective or prospective studies and were deemed to have a high overall risk of bias due to confounding. Each study included in the meta-analysis is evaluated separately in Figure 18.

## Discussion

With an emphasis on important clinical outcomes such as post-operative fracture healing, revision surgery, nonunion, and malunion, this meta-analysis synthesizes comparative information on antegrade vs retrograde intramedullary nailing (IMN) for femoral shaft and distal femur fractures in older individuals.

### Union and nonunion outcomes

Both antegrade and retrograde IMN approaches have shown good overall union rates without statistically significant differences in previous comparative investigations [20]. Retrograde and antegrade nails showed equal healing rates following the index treatment in traditional comparative cohort data, with similar nonunion incidence (about 6% in both groups) and eventual healing following additional surgeries in most patients [20].

Furthermore, when fixation is satisfactory and fracture reduction is adequate, systematic research indicates that nonunion rates are generally modest and not greatly impacted by the direction of nail entrance [23].

Our study reported that retrograde had a higher rate of union than antegrade.

### Malunion and alignment

Comparative studies of femoral shaft fractures also show that malunion rates, which indicate the quality of alignment following repair, are generally similar among procedures. In a large retrospective series, for example, malunion was documented in 11% of retrograde and 13% of antegrade instances, with no significant difference between groups [20].

However, compared with retrograde procedures, antegrade nailing may represent a slightly higher risk of loss of coronal alignment in particular fracture subgroups, possibly because of mechanical leverage and entry site biomechanics, according to some data in the larger femoral fracture literature [24].

Our study showed antegrade nailing had a higher rate of malunion and delayed union, though none of our results showed a statistically significant difference.

### Revisions and complications

In existing investigations of adult femur fractures, reoperation and revision rates – important indicators of early postoperative failure – do not seem to consistently favor one strategy over the other [25]. The general safety of both procedures for shaft fractures was highlighted by a recent systematic study that compared antegrade and retrograde treatments and found no significant difference in reoperation rates or the majority of secondary problems [25].

Interestingly, the pattern of problems seems to vary depending on the entrance method: anterior knee discomfort is more usually linked to retrograde nailing, while proximal thigh or hip pain is more frequently related to antegrade nailing [20].

Although antegrade intramedullary nailing has been associated with higher rates of malalignment or delayed union in certain fracture patterns, it may paradoxically demonstrate lower revision rates due to several biomechanical and technical factors. Antegrade nails provide a more central, load-sharing construct along the anatomical axis of the femur, which can enhance overall stability and promote sufficient callus formation even in the presence of minor malalignment. In addition, the entry point and proximal fixation allow for better control of femoral length and rotation in many shaft fractures, reducing the likelihood of gross mechanical failure requiring revision. Furthermore, delayed unions following antegrade nailing are often amenable to non-operative management or minor adjuncts (e.g., dynamization), whereas complications associated with alternative techniques may necessitate more complex reoperations [5]. These factors may explain the lower observed revision rates despite theoretical concerns regarding alignment and healing [5].

Our study showed the antegrade approach had a significantly lower rate of revisions and a lower incidence of complications after primary fixation.

### Functional outcomes and older patients

Although they are less commonly recorded, functional outcomes in geriatrics that go beyond simple radiographic recovery, such as pain, mobility, and quality of life, are crucial. There are no discernible differences between antegrade and retrograde nails in adult populations according to long-term patient-reported outcomes (such as WOMAC and VAS), indicating that both approaches can produce satisfactory functional recovery [26].

### Limitations and implications

The lack of large cohorts specifically targeting older populations (≥65 or ≥80 years) limits the generalisability of existing evidence to the management of geriatric fractures, where physiological reserve, bone quality, comorbidities, and functional demands differ substantially from those of younger cohorts. Many current studies report aggregated outcomes without adequate age stratification, potentially obscuring differences in healing rates, complication profiles, and tolerance to surgical interventions. In older patients, outcomes such as functional recovery, return to independence, mobility, and quality of life are often more clinically meaningful than radiographic union alone. Therefore, future research should prioritize adequately powered, age-stratified analyses and incorporate patient-centered outcome measures, including validated functional scores and health-related quality of life assessments, to better inform decision-making in this growing and high-risk population.

## Conclusion

Our study shows that the antegrade approach has lower revision rates despite a higher incidence of malunion and delayed union. The antegrade approach has a lower complication rate, and both approaches have similar operation times. Our authors also suggest an additional advantage of antegrade nailing in elderly patients, as this allows protection of the femoral neck. This study can serve as a basis for further clinical trials in the future.

## Data Availability

Data is available upon request.
